# Research trends and hotspots of traditional Chinese medicine for asthenopia: a comprehensive visualization and bibliometric study as of 2024

**DOI:** 10.3389/fmed.2025.1613177

**Published:** 2025-12-09

**Authors:** Tong Jin, Jia Peng, Rui Peng, Zhaoduan Hu

**Affiliations:** 1 Graduate School, Hubei University of Chinese Medicine, Wuhan, China; 2 College of Acupuncture- Moxibustion and Orthopedics-Traumatology, Hubei University of Chinese Medicine, Wuhan, China

**Keywords:** traditional Chinese medicine, asthenopia, bibliometric analysis, eye disease, CiteSpace

## Abstract

**Objective:**

To explore the preventive and therapeutic effects of traditional Chinese medicine (TCM) for asthenopia.

**Methods:**

The literatures on TCM for asthenopia published in Web of Science, PubMed, China National Knowledge Infrastructure and Wanfang from the inception of each database to December 31, 2024 were retrieved and summarized. Network cluster co-occurrence analysis and qualitative narrative methods were used for this review.

**Results:**

The related research has shown a fluctuating upward trend. The institutions that published more relevant studies were Chinese medicine universities and their affiliated hospitals. The analysis found that the research mainly focused on elucidating the treatment mechanism, optimizing the acupoint stimulation mode of external treatment, and optimizing the systematic regulation of the TCM decoction program.

**Conclusion:**

The research on TCM for asthenopia is unevenly distributed among countries and regions, and mainly concentrated in China. However, since the outbreak of COVID-19, the research on asthenopia abroad has gradually increased, which may be related to lifestyle and the development of modern electronic technology. Current research trends mainly focus on the establishment of evidence-based TCM clinical intervention programs and the establishment of a comorbidity model of asthenopia.

## 1 Introduction

Asthenopia is a common ophthalmic disorder characterized by a syndrome involving the interplay of ocular, systemic organic, psychological, and mental factors. It manifests as transient blurring near vision after prolonged use, visual haze, ocular dryness, discomfort, soreness, tearing, and systemic symptoms such as headache, dizziness, nausea, vomiting, and lethargy. Studies have revealed that visual fatigue may induce abnormalities in ocular function, musculoskeletal coordination, and immune responses (1), significantly impacting daily life and work efficiency while diminishing patients’ quality of life.

Owing to the variability of influencing factors, the incidence of asthenopia varies significantly among different job types and environments, eye habits, and age groups (2, 3). The common population with asthenopia is adults with high eye intensity, including text workers, long-term video terminal users, and close-range visual workers. Epidemiological studies (4) have shown that 23% of school-aged children, 64%–90% of computer users, and 71.3% of dry eye patients all have varying degrees of asthenopia symptoms. A research (5) found that the prevalence of asthenopia in computer workers’ video terminals can reach as high as 90%. There are reports both domestically and internationally that the incidence of asthenopia is higher in females than in males (6). With the development and progress of modern science and technology, the number of users of video terminals such as computers and mobile phones continues to increase. With the influence of factors such as a lack of outdoor sports, excessive use of electronic products, and increasing social mental pressure, the incidence rate of asthenopia has gradually increased. In addition, since the beginning of the COVID-19 pandemic, the incidence rate of asthenopia has increased significantly. Online teaching and online meetings have become normalized, and modern reading habits and learning methods, as well as entertainment and leisure activities, have increased their dependence on video terminals. Asthenopia tends toward younger age groups, with the prevalence of asthenopia among children increasing from 10% to 20% before the epidemic to 50% to 65% (7–9). Therefore, it is necessary to popularize prevention and treatment measures for asthenopia to effectively reduce the risk of its occurrence.

Traditional Chinese medicine (TCM) therapies play pivotal roles in the prevention and treatment of asthenopia, with a history spanning over two millennia and a diverse system of therapeutic modalities tailored to holistic regulation. TCM therapies can be categorized into internal and external interventions. As for internal therapies, primarily include herbal formulations that regulate the body’s internal balance based on syndrome differentiation. For example, Ziyin Runmu Tang targets liver-kidney yin deficiency-related asthenopia, while Xiaoyao San alleviates liver qi stagnation-induced symptoms. Additionally, Medicinal Cuisine—a unique TCM modality combining edible ingredients with medicinal herbs serves as a daily preventive measure by nourishing eye-related meridians. As for external therapies, encompass a wide range of non-oral interventions, including: acupuncture and moxibustion, which stimulating specific acupoints to unblock meridians and regulate qi-blood; tuina and gua sha, which relieving muscular tension around the eyes and improving local microcirculation; cupping, which using negative pressure to enhance blood perfusion in the neck and shoulder regions, indirectly alleviating ocular fatigue; acupoint catgut embedding therapy, which implanting absorbable catgut into acupoints for sustained stimulation, suitable for chronic asthenopia; qigong and tai chi, which gentle movements and breathing exercises to balance the body’s qi, reducing systemic fatigue that contributes to ocular strain.

Moreover, TCM meditation and relaxation exercises, which are important components of TCM Jing Tiao and hold significant potential for asthenopia intervention. Specifically, TCM meditation focuses on adjusting breathing rhythm and concentrating the mind on the dantian or ocular region, thereby calming the heart and soothing the liver—addressing the TCM pathogenesis of “liver qi stagnation” or “heart spirit disturbance” associated with asthenopia. Meanwhile, targeted relaxation exercises combine gentle eye movements with meditative focus, alleviating both the physical tension of extraocular muscles and the mental stress exacerbated by prolonged screen use.

Studies on internal herbal formulations for asthenopia demonstrate that leveraging TCM’s strengths, such as its holistic approach and treatment with syndrome differentiation, often yields superior clinical outcomes (10). Meta-analyses on acupuncture for asthenopia indicate its efficacy both as a standalone therapy and as an adjunct treatment, outperforming conventional interventions such as artificial tears alone (11, 12). Auricular acupoint therapy and massage have also been proven effective in alleviating symptoms of asthenopia (13, 14). TCM external therapies exhibit rigorous empirical precision in acupoint selection (15). In clinical practice, these therapies predominantly involve combination treatments, which significantly enhance efficacy while maintaining safety.

Bibliometrics allows a quantitative analysis of the created literature and the identification of key information in the relevant areas. Therapy in TCM includes a variety of treatments, and there is currently no research that provides a complete document metric analysis in this area. Therefore, to help researchers quickly understand the main content and direction of research in this field, this article summarizes and discusses information related to the treatment of asthenopia with TCM. CiteSpace is a tool for the analysis of scientific literature. CiteSpace analyzes critical information about authors, institutions and keywords in the literature. To summarize the current state of research and hot spots, we predict future trends in research and intuitively show them. Visual analysis is an analytical model that comprehensively and intuitively presents research data in the form of images and is widely used in all fields. The aim of this research is visualization, which will allow researchers to quickly understand the system of basic knowledge and the status of research in the treatment of asthenopia with TCM and determine the direction of future research.

## 2 Materials and methods

### 2.1 Data sources and search strategy

The bibliographic data were as of December 31, 2024. The search platforms for this study included the Web of Science Core Collection Database (WOS), PubMed, China National Knowledge Infrastructure (CNKI) and Wanfang. The search period spanned from the inception of each database to December 31, 2024. The search strategy was comprehensively designed to include all TCM therapeutic forms, such as herbal medicine, acupuncture, moxibustion, tuina, cupping, Gua Sha, acupoint catgut embedding, Qigong, and Tai Chi. Owing to the different retrieval methods and details of the different retrieval databases, detailed search terms and strategies are shown in Table 1.

**TABLE 1:** Search strategies.

Database	Search term	
WOS	#1	TS = (Asthenopia or Eyestrain or Visual Fatigue or Xerophthalmia or Dry Eye or Computer Vision Syndrome or video display terminal or Visual Display Terminal or Myopia or Myopias or Nearsightedness or Refractive Error or Refractive Disorder or Ametropia)
	#2	TS = (Chinese Traditional Medicine or Zhong Yi Xue or Traditional Tongue Diagnosis or Traditional Tongue Diagnoses or Traditional Tongue Assessment)
	#3	TS = (Chinese Drugs, Plant or Chinese Herbal Drugs or Chinese Plant Extracts)
	#4	TS = (moxibustion or Acupuncture or Pharmacopuncture or Pharmacoacupuncture or Acupuncture Therapy or Acupuncture Treatment or Acupotomy)
	#5	TS = (Massage or Zone Therapy or Acupuncture Point or Acupoints or Acupoint or Tuina or Cupping or Gua Sha or Scraping or Acupoint Catgut Embedding or Qigong or Tai Chi or Medicinal Cusisine)
	#6	#2 or #3 or #4 or #5
	#7	#1 and #6
		Refined By: Publication Years: 2024 or 2023 or 2022 or 2021 or 2020 or 2019 or 2018 or 2017 or 2016 or 2015 or 2014. Document Types: Article or Review Article. Languages: English. Open Access: All Open Access.
PubMed	#1	”Asthenopia”[Mesh]
	#2	(((((Eyestrain[Title/Abstract])) OR (Fatigue, Visual[Title/Abstract])) OR (Visual Fatigue[Title/Abstract])) OR (Eye Fatigue[Title/Abstract])) OR (Fatigue, Eye[Title/Abstract])
	#3	”Dry Eye Syndromes”[Mesh]
	#4	((((((((((Dry Eye Syndrome[Title/Abstract])) OR (Dry Eye Disease[Title/Abstract])) OR (Dry Eye Diseases[Title/Abstract])) OR (Dry Eye[Title/Abstract])) OR (Dry Eyes[Title/Abstract])) OR (Evaporative Dry Eye Disease[Title/Abstract])) OR (Evaporative Dry Eye[Title/Abstract])) OR (Dry Eye, Evaporative[Title/Abstract])) OR (Evaporative Dry Eyes[Title/Abstract])) OR (Evaporative Dry Eye Syndrome[Title/Abstract])
	#5	”Xerophthalmia”[Mesh]
	#6	((((Computer Vision Syndrome[Title/Abstract]) OR (video display terminal[Title/Abstract])) OR (video display terminal Syndrome[Title/Abstract])) OR (Visual Display Terminal[Title/Abstract])) OR (Visual Display Terminal Syndrome[Title/Abstract])
	#7	#1 OR #2 OR #3 OR #4 OR #5 OR #6
	#8	”Medicine, Chinese Traditional”[MeSH]
	#9	((((((((((((Zhong Yi Xue[Title/Abstract])) OR (Traditional Medicine, Chinese[Title/Abstract])) OR (Chinese Traditional Medicine[Title/Abstract])) OR (Traditional Chinese Medicine[Title/Abstract])) OR (Chinese Medicine, Traditional[Title/Abstract])) OR (Traditional Tongue Diagnosis[Title/Abstract])) OR (Tongue Diagnoses, Traditional[Title/Abstract])) OR (Tongue Diagnosis, Traditional[Title/Abstract])) OR (Traditional Tongue Diagnoses[Title/Abstract])) OR (Traditional Tongue Assessment[Title/Abstract])) OR (Tongue Assessment, Traditional[Title/Abstract])) OR (Traditional Tongue Assessments[Title/Abstract])
	#10	”Drugs, Chinese Herbal”[MeSH]
	#11	((((((Chinese Drugs, Plant[Title/Abstract])) OR (Chinese Herbal Drugs[Title/Abstract])) OR (Herbal Drugs, Chinese[Title/Abstract])) OR (Plant Extracts, Chinese[Title/Abstract])) OR (Chinese Plant Extracts[Title/Abstract])) OR (Extracts, Chinese Plant[Title/Abstract])
	#12	(“Acupuncture”[MESH]) OR (Pharmacopuncture[Title/Abstract])
	#13	”Acupuncture Therapy”[Mesh]
	#14	((((((((((Acupuncture Treatment[Title/Abstract])) OR (Acupuncture Treatments[Title/Abstract])) OR (Treatment, Acupuncture[Title/Abstract])) OR (Therapy, Acupuncture[Title/Abstract])) OR (Pharmacoacupuncture Treatment[Title/Abstract])) OR (Treatment, Pharmacoacupuncture[Title/Abstract])) OR (Pharmacoacupuncture Therapy[Title/Abstract])) OR (Therapy, Pharmacoacupuncture[Title/Abstract])) OR (Acupotomy[Title/Abstract])) OR (Acupotomies[Title/Abstract])
	#15	(“moxibustion”[MESH]) OR (Moxabustion[Title/Abstract])
	#16	”Massage”[Mesh]
	#17	((((((((Zone Therapy[Title/Abstract])) OR (Therapies, Zone[Title/Abstract])) OR (Zone Therapies[Title/Abstract])) OR (Therapy, Zone[Title/Abstract])) OR (Massage Therapy[Title/Abstract])) OR (Massage Therapies[Title/Abstract])) OR (Therapies, Massage[Title/Abstract])) OR (Therapy, Massage[Title/Abstract])
	#18	”Acupuncture Points”[Mesh]
	#19	(((((Acupuncture Point[Title/Abstract])) OR (Point, Acupuncture[Title/Abstract])) OR (Points, Acupuncture[Title/Abstract])) OR (Acupoints[Title/Abstract])) OR (Acupoint[Title/Abstract])
	#20	”Cupping Therapy”[Mesh]
	#21	((((Cupping Therapies[Title/Abstract]) OR (Therapy, Cupping[Title/Abstract])) OR (Cupping Treatment[Title/Abstract])) OR (Cupping Treatments[Title/Abstract])) OR (Treatment, Cupping[Title/Abstract])
	#22	”Qigong”[Mesh]
	#23	(Ch’i Kung[Title/Abstract]) OR (Qi Gong[Title/Abstract])
	#24	#8 OR #9 OR #10 OR #11 OR #12 OR #13 OR #14 OR #15 OR #16 OR #17 OR #18 OR #19 OR #20 OR #21 OR #22 OR #23
	#25	#7 AND #24
		Filters: Full text, English, from 1974 to 2024
CNKI	#1	TKA = ('视疲劳'+'眼疲劳'+'视觉疲劳'+'眼睛疲劳'+'眼部疲劳'+'视频终端综合症'+'视频终端综合征'+'干眼性视疲劳'+'干眼疲劳')
	#2	TKA = ('中医'+'中药'+'中医药'+'散'+'汤'+'剂'+'丸'+'针灸'+'针'+'针刺'+'灸'+'手法'+'按摩'+'推拿'+'刮痧'+'耳穴'+'中医外治'+'穴位'+'经络'+'拔罐'+'气功'+'太极拳'+'埋线')
	#3	#1 AND #2
		资源范围：总库-中文；时间范围：发表时间：截至到2024-12-31；检索范围：学术期刊
Wanfang	#1	(题名或关键词:(视疲劳 or 眼疲劳 or 视觉疲劳 or 眼睛疲劳 or 眼部疲劳 or 视频终端综合症 or 视频终端综合征 or 干眼性视疲劳 or 干眼疲劳) and 题名或关键词:(中医 or 中药 or 中医药 or 散 or 汤 or 剂 or 丸 or 针灸 or 针 or 针刺 or 灸 or 手法 or 推拿 or 按摩 or 埋线 or 拔罐 or 刮痧 or 耳穴 or 穴位敷贴 or 中医外治 or 穴位 or 经络 or 气功 or 太极拳)) and 出版时间:[* TO 2024-12-31}
		文献类型：期刊论文；获取范围：有全文；学科分类：医药、卫生；语种：中文

### 2.2 Article screening criteria

#### 2.2.1 Inclusion criteria

The inclusion criteria were as follows: (i) articles related to TCM treatment for asthenopia; (ii) journal articles; and (iii) articles containing complete information, including publication date, author, institution, and keywords.

#### 2.2.2 Exclusion criteria

The exclusion criteria were as follows: (i) studies unrelated to TCM treatment for asthenopia; (ii) repeatedly published articles; (iii) retracted publications; and (iv) meeting documents, news, advertisements, patents, and popular science articles.

#### 2.2.3 Data entry and specification

Data from WOS and PubMed were exported in plain text format with complete records and references. Data from CNKI and Wanfang were presented in “refworks” format and converted to the download_***.txt format for use. CiteSpace software (V6.4. R1) to examine and organize the articles. The data cleaning work was completed after the final file was confirmed.

Data cleaning principles: (i) Specify the name of the institution. For different names of the same institution, the currently widely accepted specification name is used. The same institution does not subdivide departments, colleges, divisions, etc. For example, the “School of Chinese Material Medical of Beijing University of Traditional Chinese Medicine,” the “School of Acupuncture and Massage of Beijing University of Traditional Chinese Medicine” and the “First Clinical Medical College of Beijing University of Traditional Chinese Medicine” are unified into the “Beijing University of Traditional Chinese Medicine.” The “Rehabilitation Department of the First Affiliated Hospital of Guangxi University of Traditional Chinese Medicine” and the “Massage Department of the First Affiliated Hospital of Guangxi University of Traditional Chinese Medicine” were unified as the “First Affiliated Hospital of Guangxi University of Traditional Chinese Medicine”; (ii) Merge keywords. For keywords of great significance to the interpretation of the atlas, different expressions of the same meaning are unified into normative terms or common expressions, such as “asthenopia,” “eye fatigue” and “visual fatigue,” which are unified as “asthenopia.”

### 2.3 Data analysis

CiteSpace and Microsoft Excel software were used for bibliometric analysis of the literature, providing universal year links and citation cocitation count maps to analyze global bibliographic material outputs and trends. CiteSpace is a scientific literature analysis platform that employs bibliometric methods, data mining algorithms, and knowledge domain visualization techniques to detect emerging research fronts and map disciplinary development trends through interactive visual representations. The data are imported into CiteSpace, and the following parameters are set to achieve optimal results: time partition, as of 2024; time slice, “1”; and node type, which is configured as author, country, institution, or keywords. In this way, CiteSpace generated collaborative networks, coreferenced reference networks, and the strongest burst keywords. A network diagram consists of nodes, links, and colors. Nodes represent elements such as countries, regions or institutions, whereas larger nodes indicate a larger number of publications or a higher co-occurrence frequency. The bright rings around the node indicate the year of the node and are accompanied by color illustrations for reference. The links between nodes symbolize the co-occurrence trends in the same publication, and the thinner the links are, the lower the frequency of co-occurrence trends. The centrality of network nodes is calculated to identify key nodes. High centrality indicates that nodes play a crucial role in connecting different network parts and signaling important transitions or critical articles. Keyword clustering analysis revealed the main themes, trends, and significant changes in the research topic.

## 3 Results

### 3.1 Annual publication analysis

**FIGURE 1:**
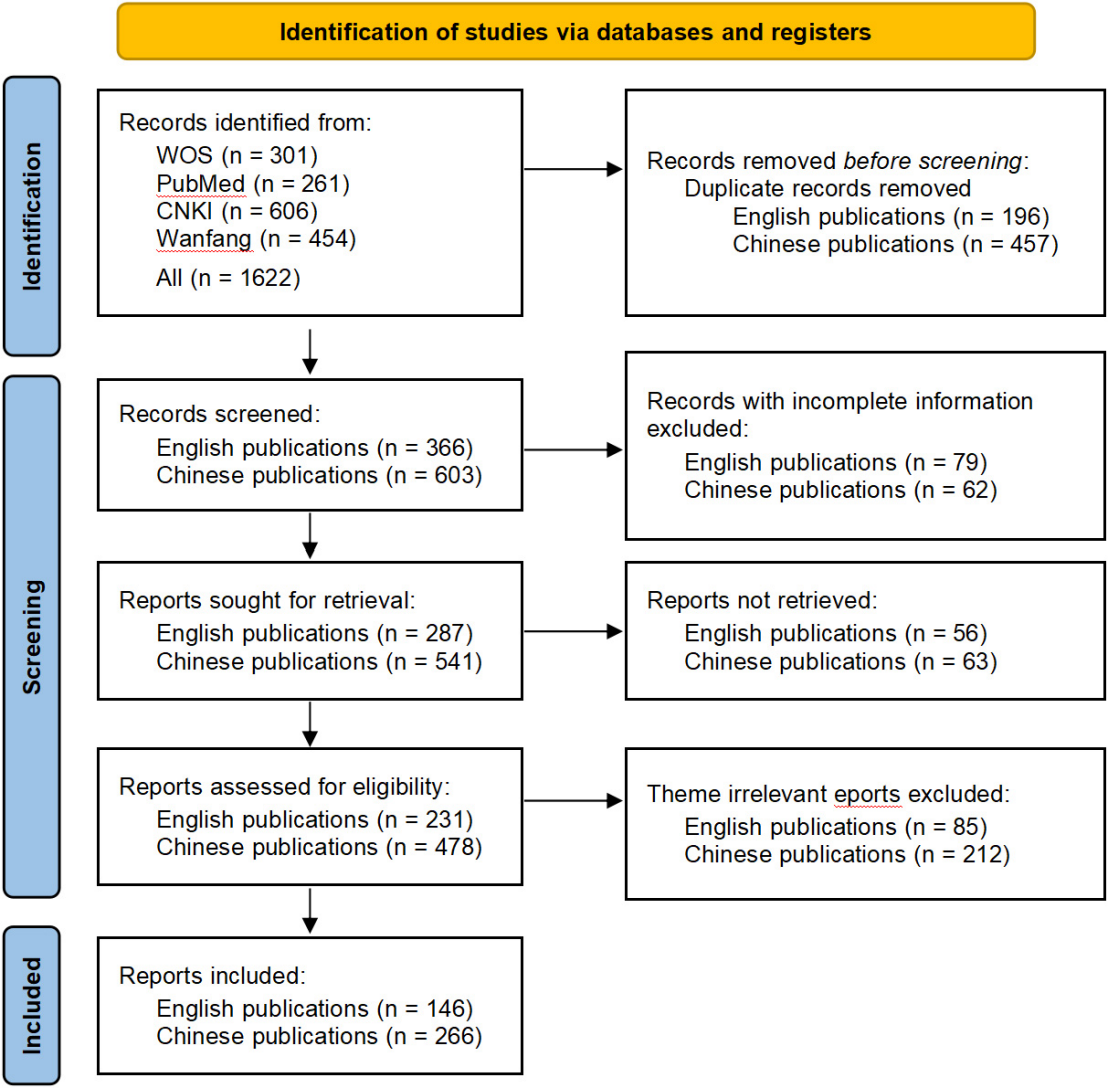
Flowchart of the inclusion and exclusion criteria for publications.

**FIGURE 2:**
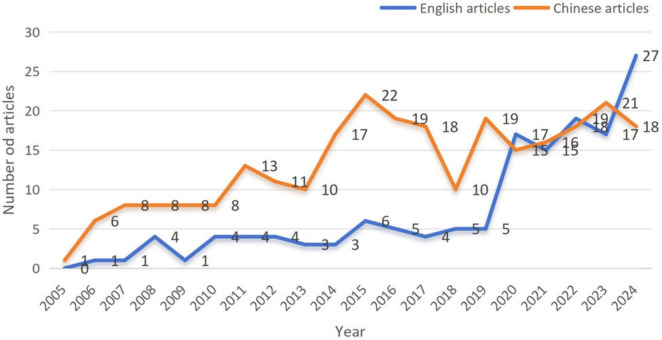
Annual articles on TCM treatment for asthenopia.

Figure 1 delineates the literature screening workflow for TCM treatment of asthenopia. The systematic retrieval yielded 412 eligible publications spanning as of 2024, comprising 146 English-language articles and 266 Chinese-language articles. The temporal publication patterns are visualized in Figure 2 Longitudinal analysis of English-language publications reveals a consistent growth trajectory from the inception of each database through 2020, attaining a peak of 27 publications in 2024. Notably, the post-2019 period witnessed an accelerated publication frequency. This is potentially attributable to pandemic-induced lifestyle changes during the COVID-19 global health crisis that exacerbated the prevalence of asthenopia. The surge underscores the field’s growing societal relevance. Chinese-language publications exhibited cyclical variation, achieving maximum annual output (*n* = 22) in 2015. Despite interannual fluctuations, the cumulative output demonstrates sustained growth, expanding from a baseline of 1 publication in 2005 to 18 in 2024. The synthesis of both databases revealed a 45-fold increase in annual publication volume over the study period (2005: *n* = 1 vs. 2024: *n* = 45), reflecting intensified scholarly engagement with TCM-based asthenopia therapeutics. This compound growth pattern signals the field’s transition from niche investigation to mainstream research.

### 3.2 Author publication analysis

**FIGURE 3:**
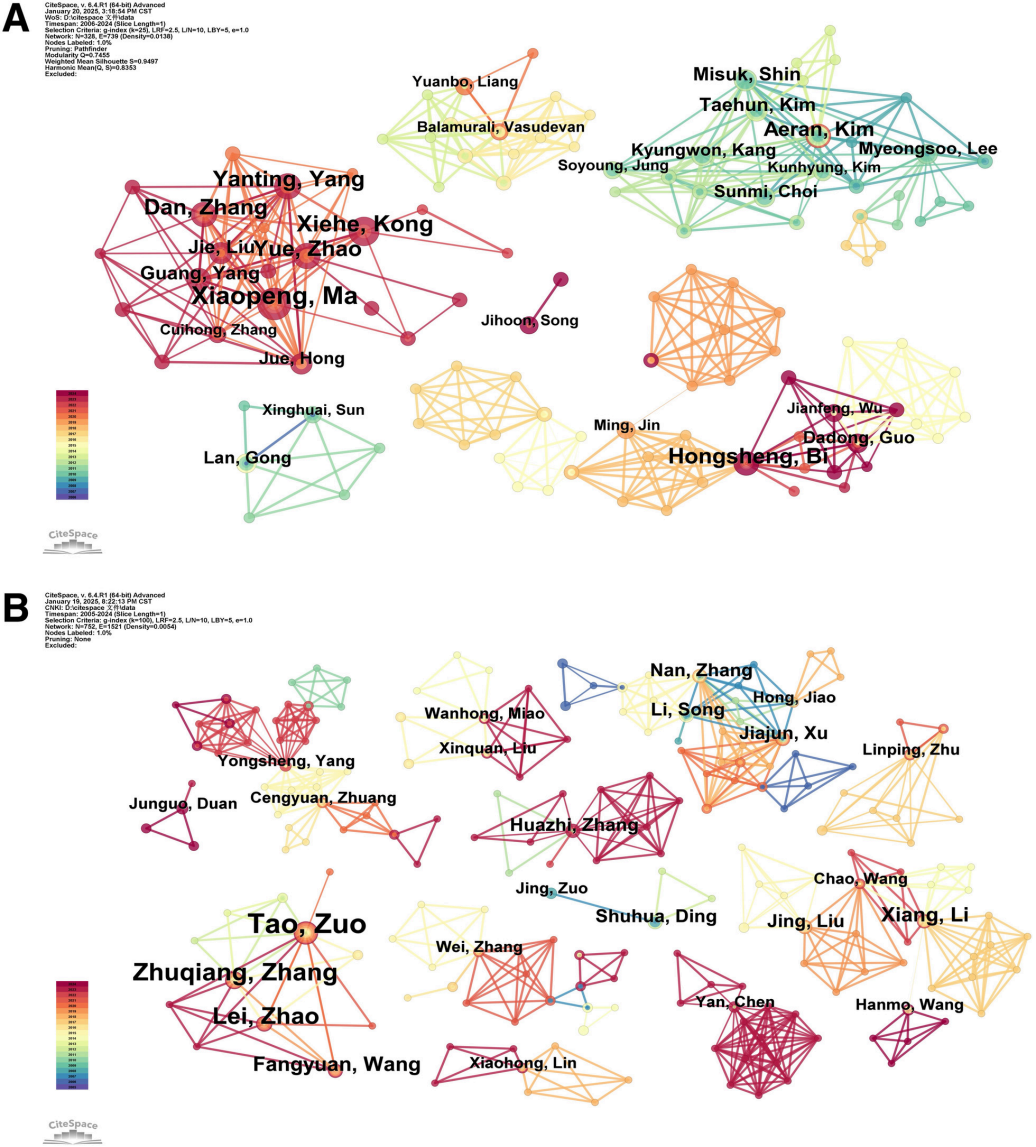
Network map of authors. (A) English literature. (B) Chinese literature.

Figure 3 presents the author collaboration networks generated through CiteSpace software. Table 2 lists the top 10 authors by publication volume. The analysis of author collaboration networks highlights the most active and impactful researchers in this field, while also revealing structural characteristics of research teams. This study applies Price’s law to identify core authors. According to Price’s Law, the threshold for core authorship is calculated as: $\text{M}=0.749\times \sqrt{{\text{N}}_{\text{max}}}$, where M represents the minimum publication count required to qualify as a core author, and Nmax denotes the highest number of publications by a single author during the analysis period.

**TABLE 2:** The top 10 authors publishing articles.

No.	English literature		-	No.	Chinese literature	
	n	Author			n	Author
1	11	Xiaopeng Ma		1	11	Tao Zuo
2	8	Xiehe Kong		2	8	Zhuqiang Zhang
2	8	Yanting Yang		3	7	Lei Zhao
2	8	Yue Zhao		4	5	Fangyuan Wang
5	7	Hongsheng Bi		4	5	Xiang Li
5	7	Dan Zhang		6	4	Nan Zhang
7	6	Jue Hong		6	4	Huazhi Zhang
7	6	Aeran Kim		6	4	Jiajun Xu
7	6	Taehun Kim		6	4	Li Song
7	6	Jie Liu		6	4	Jing LIu
7	6	Misuk Shin		6	4	Shuhua Ding

In the English literature collaboration network (Figure 3A), Xiaopeng Ma (Nmax = 11) is the core hub with the highest publication volume. Substituting into the formula yielded M≈2.48. Therefore, authors with 3 or more publications were classified as core authors, and a total of 17 core authors were identified, accounting for 65.8% of the total number of publications. This proportion significantly exceeds the 50% threshold stipulated by Price’s Law, confirming the establishment of a robust core author group in English-language TCM asthenopia research. Among them, Xiaopeng Ma formed a closely connected research cluster with Xiehe Kong, Yanting Yang, and Yue Zhao (all with *n* = 8). This cluster focuses on conducting clinical trials on electro-acupuncture therapy. In contrast, isolated groups (e.g., the cluster led by Myeongsoo Lee, *n* = 3) have limited collaboration with core authors, indicating regional or thematic research isolation, for instance, focusing solely on a certain therapy without integrating it with other TCM practices.

For Chinese literature (Figure 3B), Tao Zuo (Nmax = 7) is the central figure, resulting in M≈2.65, and 16 authors met the ≥3 publication criterion for core status. Their collective output constituted 22.2% of the total publications, failing to meet the 50% threshold required by Price’s Law. This disparity indicates an underdeveloped core author network in Chinese-language TCM asthenopia studies. Among them, Tao Zuo collaborated closely with Zhuqiang Zhang (*n* = 8) and Lei Zhao (*n* = 7) to form a dominant cluster focusing on acupoint therapy and herbal decoctions . Isolated authors (e.g., Nan Zhang, Hong Jiao, both with *n* = 4) lack connections with the core cluster, reflecting insufficient academic collaboration among some Chinese research teams, which possibly due to regional resource disparities or independent research focuses.

### 3.3 National and regional publication analysis

**FIGURE 4:**
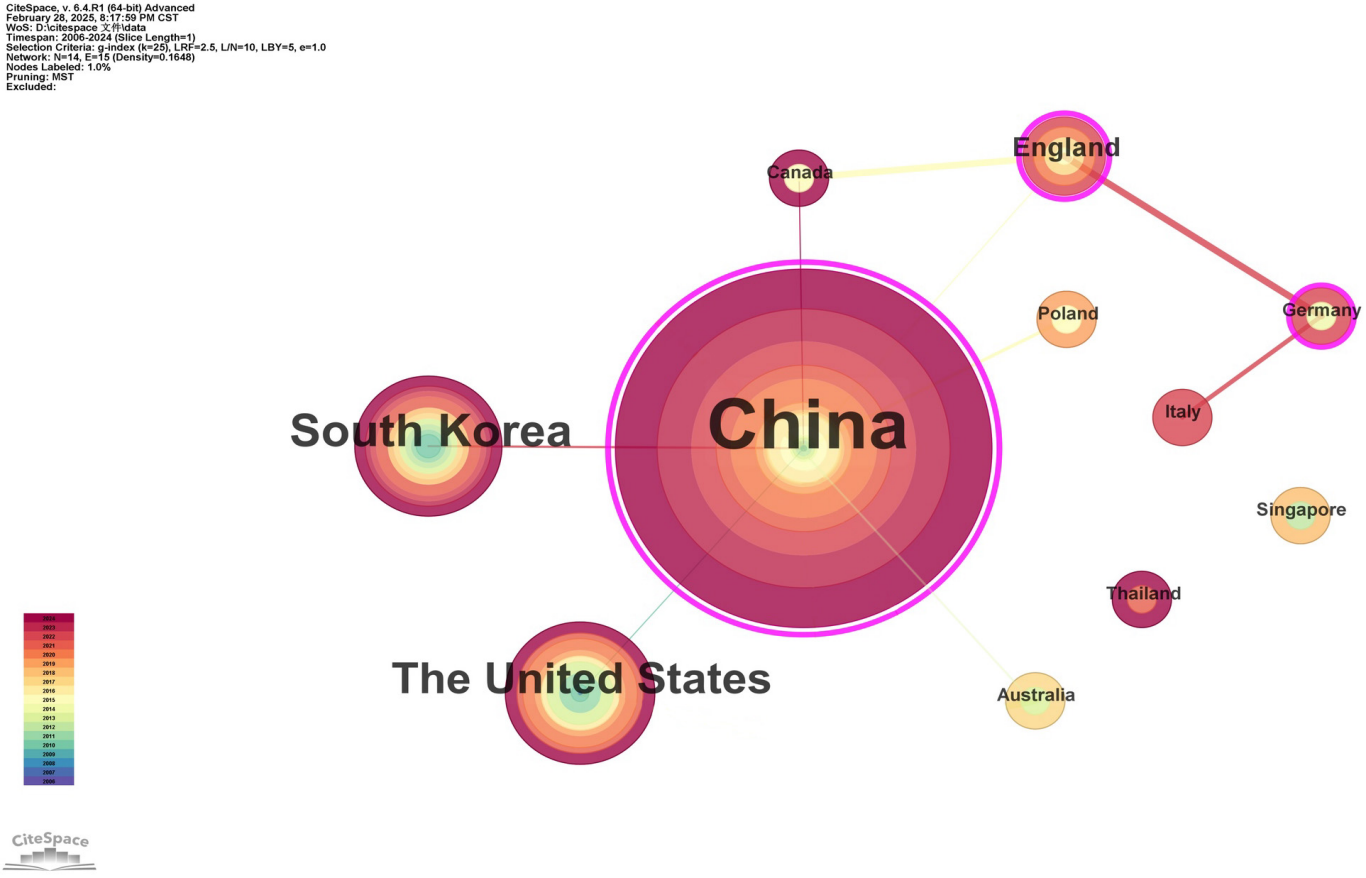
Network map of national and regional network collaborations (China includes Taiwan, Macao and Hong Kong regions).

A bibliometric network analysis was performed on international collaboration patterns in TCM-based asthenopia research via English-language literature, with visualized outputs presented in the national/regional cooperation map (Figure 4) and ranked publication metrics (Table 3). Betweenness centrality values, which quantify nodes’ structural importance in knowledge exchange networks, revealed critical contributors. Nodes exceeding the centrality threshold of ≥0.1 were identified as pivotal hubs. In the analysis, China (including Taiwan, Hong Kong, and Macao) emerged as the dominant contributor, producing 99 publications (67.8% of total output) with the highest betweenness centrality (1.39), reflecting its central role in bridging global research efforts, such as collaborating with South Korea on acupuncture mechanisms and the USA on evidence-based TCM. This predominance aligns with TCM’s institutionalization in China’s healthcare system. In contrast, other nations demonstrated limited engagement and fragmented collaboration. The country with the second highest publication volume (South Korea) contributed only 16 publications (11.0%), focusing primarily on acupoint therapy with weak connections to non-Asian countries. The country with the second highest centrality (England) had a subthreshold centrality of 0.46, with research limited to “systematic reviews” of TCM efficacy but no original clinical studies. Most Western countries (e.g., Canada, Italy, Australia) formed small, isolated clusters (≤2 publications each). This fragmented collaboration network underscores the unmet potential for international synergy, which may be related to three factors: (1) Geographical differences: Most collaborations occur within Asia, with cross-border cooperation in Europe/North America hindered by distance; (2) Language barriers: Standardized TCM terminology lacks consistent English translations, creating comprehension barriers for foreign scholars; (3) Asymmetric resources: China has relatively abundant traditional Chinese medicine resources, whereas foreign countries focus more on research related to Western medicine.

**TABLE 3:** The top 10 countries and regions in terms of volume and top 3 centrality of publications.

No.	n	Countries and territories	Year	-	No.	Centrality	Countries and territories	Year
1	99	China	2007		1	1.39	China	2007
2	15	South Korea	2009		2	0.46	England	2015
3	13	The United States	2008		3	0.26	Germany	2014
4	4	England	2015					
5	2	Singapore	2012					
5	2	Thailand	2021					
5	2	Canada	2015					
5	2	Poland	2015					
5	2	Italy	2022					
5	2	Australia	2013					
5	2	Germany	2014					

Data of Taiwan, Macao and Hong Kong regions are included in China, in line with the one-China principle.

### 3.4 Institutional publication analysis

**FIGURE 5:**
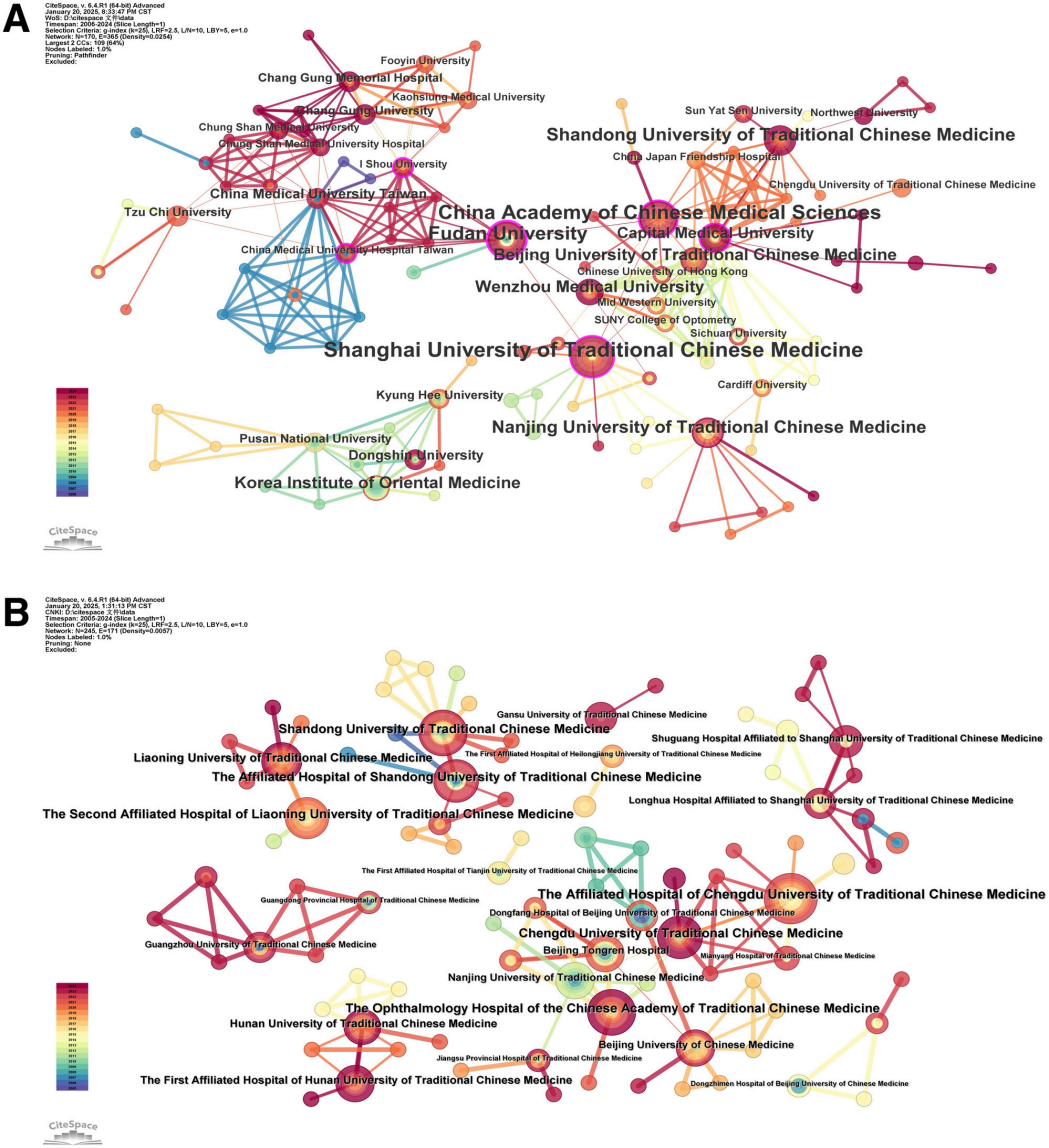
Institutional collaboration network map. (A) English literature. (B) Chinese literature.

An institutional collaboration network was constructed through CiteSpace bibliometric analysis, with visual outputs comprising a node-link diagram of institutions (Figure 5) and a ranked list of the top 10 productive institutions by publication output (Table 4). The diameter of nodes in the network correlates with publication volume, and link thickness reflects the frequency of cooperation between institutions.

**TABLE 4:** Top 10 institutions publishing articles.

No.	English literature		-	No.	Chinese literature	
	n	Institution			n	Institution
1	17	Shanghai University of Traditional Chinese Medicine		1	12	The Ophthalmology Hospital of the Chinese Academy of Traditional Chinese Medicine
2	15	China Academy of Chinese Medical Sciences		2	11	The Affiliated Hospital of Chengdu University of Traditional Chinese Medicine
3	12	Fudan University		2	11	The Second Affiliated Hospital of Liaoning University of Traditional Chinese Medicine
4	10	Shandong University of Traditional Chinese Medicine		4	10	Shandong University of Traditional Chinese Medicine
5	9	Nanjing University of Traditional Chinese Medicine		5	9	Chengdu University of Traditional Chinese Medicine
6	8	Beijing University of Traditional Chinese Medicine		5	9	The Affiliated Hospital of Shandong University of Traditional Chinese Medicine
6	8	Korea Institute of Oriental Medicine		7	8	Liaoning University of Traditional Chinese Medicine
6	8	Wenzhou Medical University		8	7	Hunan University of Traditional Chinese Medicine
9	7	Capital Medical University		8	7	The First Affiliated Hospital of Hunan University of Traditional Chinese Medicine
10	5	China Medical University Taiwan		10	6	Beijing Tongren Hospital, Capital Medical University
10	5	Dongshin University		10	6	Beijing University of Chinese Medicine
				10	6	Nanjing University of Traditional Chinese Medicine

Table 4 revealed that the top 10 institutions with the most published articles are mainly universities of TCM from China and its affiliated hospitals, forming core collaboration clusters while some regional/international institutions remain isolated. In terms of English publications (Figure 5A), Shanghai University of Traditional Chinese Medicine (*n* = 17) is the core hub, collaborating closely with the China Academy of Chinese Medical Sciences (*n* = 15) and Fudan University (*n* = 12) to form a research alliance focusing on: (1) evidence-based TCM; (2) external therapy innovation; and (3) mechanism exploration. Isolated institutions (e.g., Dongshin University, *n* = 5; Mid Western University, *n* = 3) have limited cross-institutional cooperation, focusing solely on regional research (e.g., “auricular acupressure for computer workers” in South Korea) without integrating into the core alliance. In terms of Chinese publications (Figure 5B), the Ophthalmology Hospital of the Chinese Academy of Traditional Chinese Medicine (*n* = 12) is the central institution, collaborating with the Affiliated Hospital of Chengdu University of Traditional Chinese Medicine (*n* = 11) and the Second Affiliated Hospital of Liaoning University of Traditional Chinese Medicine (*n* = 10) on: (1) TCM syndrome differentiation for asthenopia; (2) clinical application of external therapies; and (3) herbal decoction optimization. Isolated institutions (e.g., Gansu University of Traditional Chinese Medicine, *n* = 3; Mianyang Hospital of Traditional Chinese Medicine, *n* = 2) reflect regional disparities in research collaboration intensity—likely due to limited clinical resources, small sample sizes for trials, and the lack of access to core research networks. These institutions represent a key force in TCM research, with abundant resources and professional research teams, and have achieved greater success in related research fields. The large number of articles published by universities of TCM provide strong support for the preservation and development of TCM.

### 3.5 Keyword network analysis

#### 3.5.1 Keyword co-occurrence

**FIGURE 6:**
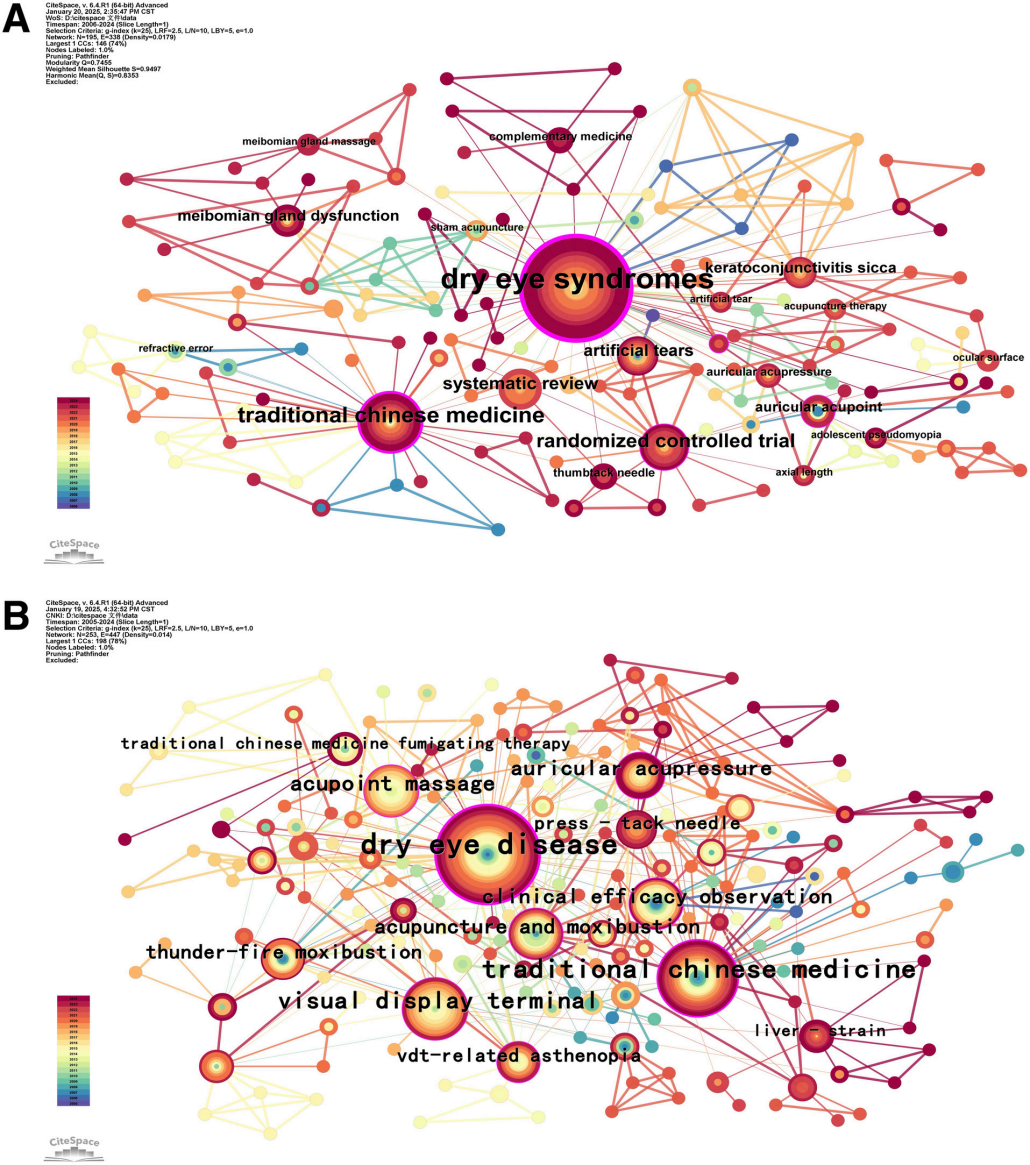
Visualization of keyword co-occurrence. (A) English literature. (B) Chinese literature.

A keyword co-occurrence map (Figure 6) is generated, and the requencies of keywords (Table 5) are ranked via CiteSpace software. The keywords and their network diagrams succinctly reflect the research topics, which helps identify the hotspots in this field. Among them, higher frequencies indicate more intensive research in the field, whereas higher concentrations indicate the importance of research in the field.

**TABLE 5:** The top 10 frequency-ranking keywords.

No.	English literature			-	Chinese literature		
	Frequency	Centrality	Keyword		Frequency	Centrality	Keyword
1	68	0.064	Dry eye syndromes		139	0.130	Asthenopia
2	49	0.046	Acupuncture		61	0.057	Dry eye disease
3	24	0.022	Myopia		38	0.036	Traditional Chinese medicine
4	22	0.021	Traditional Chinese medicine		34	0.032	Acupuncture
5	15	0.014	Randomized controlled trial		29	0.027	Visual display terminal
6	13	0.012	Systematic review		19	0.018	Acupoint massage
7	12	0.011	Electroacupuncture		17	0.016	Auricular acupressure
8	11	0.010	Meta-analysis		16	0.015	Acupuncture and moxibustion
9	10	0.009	Protocol		16	0.015	Clinical efficacy observation
10	9	0.008	Artificial tears		14	0.013	Thunder-fire moxibustion

By analyzing the keywords, we can conclude that the high-frequency keywords in English literature are “dry eye syndrome,” “acupuncture,” “myopia,” “randomized controlled trial,” and “system review.” These reflect a focus on evidence-based TCM, comorbidity, and external therapy. High-frequency keywords in the Chinese literature included “dry eye disease,” “acupuncture,” “visual display terminal,” “acupoint massage,” and “auricular acupressure.” These reflect a focus on TCM syndrome differentiation, video terminal-related asthenopia, and acupoint therapy.

In summary, these results can be divided into three categories: eye diseases, TCM prevention and treatment, and research methods.

#### 3.5.2 Keyword clustering

**FIGURE 7:**
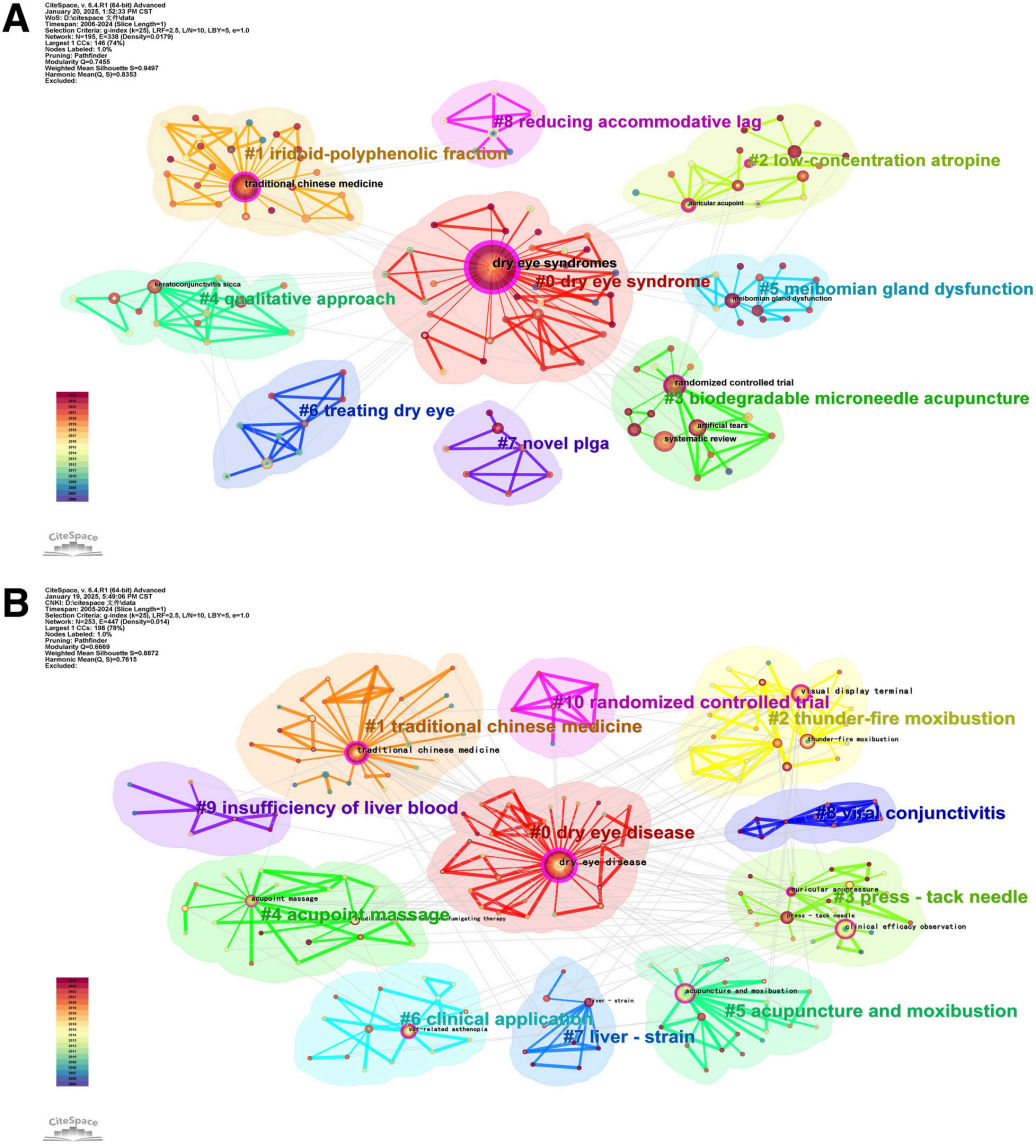
Map of keyword clustering. (A) English literature. (B) Chinese literature.

A keyword clustering map (Figure 7) was generated via CiteSpace software. On the basis of the keyword co-occurrence network, the logarithmic likelihood ratio algorithm was used to obtain the Q-cluster module value and S-cluster contour index value, where *Q* > 0.3 indicated effective clustering, and the larger the *Q*-value was, the better the network clustering effect. When *S* > 0.7, the results of the cluster analysis were valid, and the closer the *S*-value was to 1, the greater the uniformity of the network. In our study, for English literature, *Q* = 0.7455 and *S* = 0.9497. For Chinese literature, *q* = 0.669, and *s* = 0.8872. The above values indicated that clustering was meaningful and that the results were reasonable.

Analyze the clustering mapping of keywords in English literature (Figure 7A), the 8 clusters reflect three core research directions. (1) Mechanism exploration: #1 iridoid-polyphenolic fraction (focus on anti-inflammatory/antioxidant effects of herbal components), #7 novel PLGA (drug delivery systems for TCM), #8 reducing accommodative lag (acupuncture’s regulation of ocular accommodation); (2) Comorbidity research: #0 dry eye syndrome, #5 meibomian gland dysfunction, #6 treating dry eye (asthenopia combined with ocular surface diseases); (3) Therapy innovation: #2 low-concentration atropine (combination of TCM and Western medicine), #3 biodegradable microneedle acupuncture (technological upgrade of external therapy); and Research methods: #4 qualitative approach (qualitative analysis of TCM clinical experience).

Analyze the clustering mapping of keywords in Chinese literature (Figure 7B), the 10 clusters reflect three core research directions. (1) TCM intervention application: #2 thunder-fire noxibustion, #3 press-tack needle, #4 acupoint massage, #5 acupuncture and moxibustion (diversified external therapies); (2) Comorbidity research: #0 dry eye disease, #8 viral conjunctivitis (asthenopia combined with common ocular diseases); (3) TCM mechanism research: #7 liver-strain, #9 insufficiency of liver blood (pathogenesis based on TCM zang-fu theory); and Research directions: #6 clinical application, #10 randomized controlled trial (focus on clinical translation of TCM).

**FIGURE 8:**
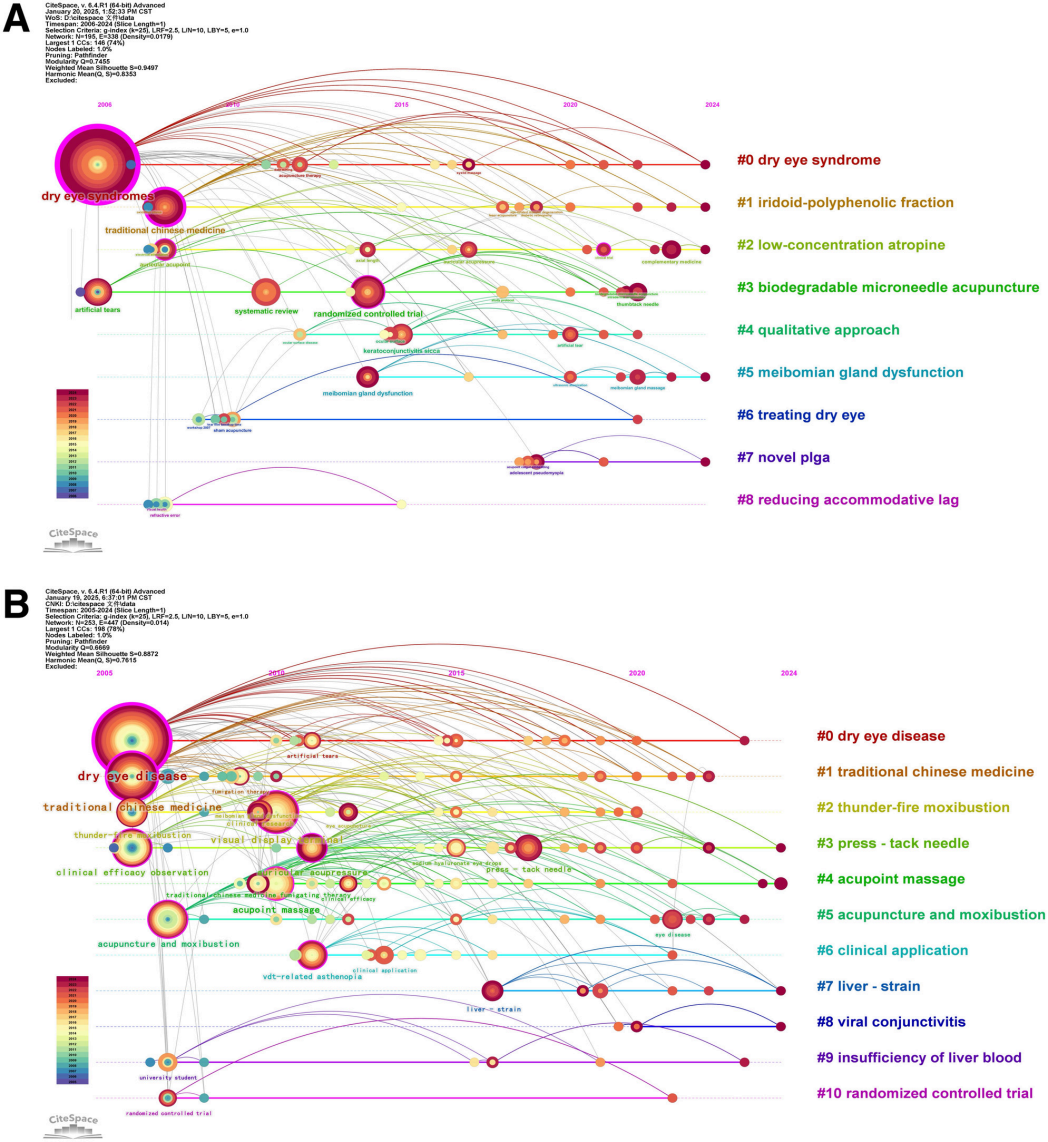
Timeline diagram of keywords. (A) English literature. (B) Chinese literature.

A timeline diagram (Figure 8) clearly displays the time range covered by each research hotspot, revealing the connections between different research hotspots, thus revealing the changes in the research hotspots of TCM treatment for asthenopia in the past years. Early stage (2005–2010): The field focused on basic TCM interventions, refractive error, and visual health, with limited mechanistic research. Mid-stage (2011–2018): Research shifted to clinical efficacy verification (e.g., “randomized controlled trial” in both literatures, “acupuncture and moxibustion” as a burst keyword in Chinese literature with intensity = 2.92), and the combination of TCM with modern technology (e.g., “visual display terminal” research). Recent (2019–2024): The focus moved to precision therapy and mechanism exploration (e.g., “biodegradable microneedle acupuncture” in English literature, “liver strain” in Chinese literature with burst intensity = 1.93), reflecting a shift from empirical practice to evidence-based and technologically integrated research.

#### 3.5.3 Keyword bursts

**FIGURE 9:**
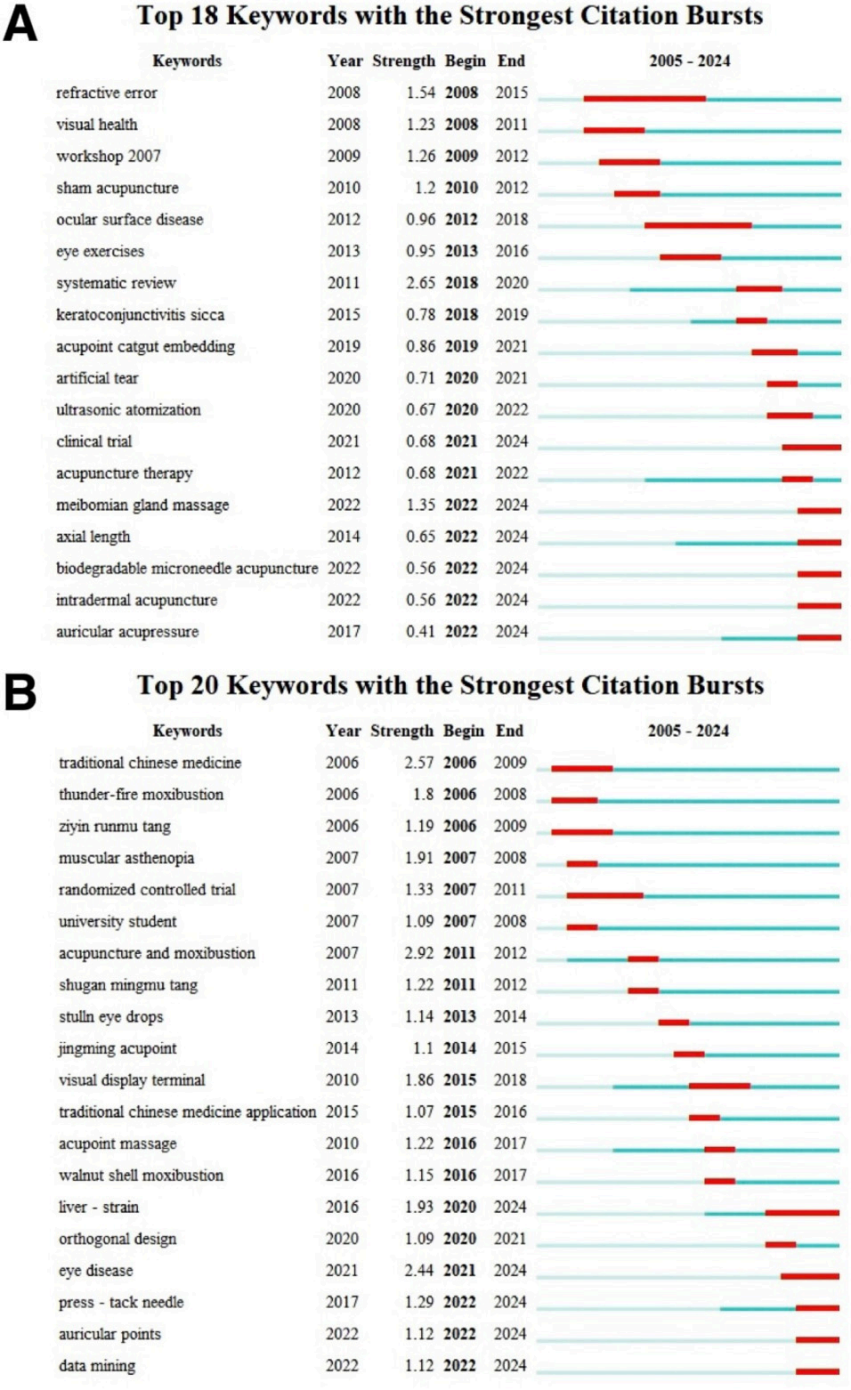
Keywords with the strongest citation bursts. (A) English literature. (B) Chinese literature.

A visual mapping of keyword bursting (Figure 9) is generated via CiteSpace software. Analyzing the emergence of keywords means identifying words with frequent occurrence and words that have rapidly increased in frequency. The blue line represents the time interval, and the red line represents the time period when the keyword bursts. In our research, keywords with strengths >2 had a significant effect on the development of the research field.

As for English literature (Figure 9A), the most persistent keyword is “refractive error” (2008–2015, burst intensity = 1.54), reflecting early focus on asthenopia’s association with refractive abnormalities. The strongest burst keyword is “systematic review” (2018–2020, intensity = 2.65), indicating a shift to evidence synthesis of TCM efficacy. Recent bursts (2022–2024) includes “biodegradable microneedle acupuncture,” “intradermal acupuncture,” and “meibomian gland massage,” reflecting technological innovation in external therapy and focus on ocular surface microenvironment. The research stage can be divided into three phases. Early phase (2005–2010) focus on basic epidemiological and etiological research, such as “refractive error,” “visual health,” and “ocular surface disease.” Middle phase (2011–2018) focus on clinical intervention verification and evidence synthesis, including “eye exercise,” “systematic review,” “keratoconjunctivitis sicca,” and “acupoint catgut embedding.” Late phase (2019–2024) focus on the combination of TCM and Western medicine, precision external therapy, containing “artificial tear,” “ultrasonic atomization,” “clinical trial,” and “acupuncture therapy.”

As for Chinese literature (Figure 9B), the most persistent keywords are “randomized controlled trial” (2007–2011, intensity = 1.33), and “liver strain” (2020–2024, intensity = 1.93), reflecting long-term focus on clinical evidence and TCM pathogenesis. The strongest burst keyword is “acupuncture and moxibustion” (2011–2012, intensity = 2.92), indicating recognition of external therapy’s core role. Recent bursts (2021–2024) includes “press-tack needle,” “auricular points,” and “data mining”, reflecting precision acupoint therapy and big data-driven TCM research. The research stage can be divided into three phases. Early phase (2005–2010) focus on basic TCM intervention and syndrome differentiation, containing “traditional Chinese medicine,” “thunder-fire moxibustion,” “ziyin runmu tang,” and “muscular asthenopia.” Middle phase (2011–2018) focus on external therapy optimization and video terminal-related asthenopia, containing “acupuncture and moxibustion,” “shugan mingmu tang,” “jingming acupoint,” “visual display terminal.” Late phase (2019–2024) focus on TCM mechanism deepening and comorbidity model research, for “liver strain,” “orthogonal design,” “eye disease,” “data mining.”

## 4 Discussion

### 4.1 General information

The bibliometric analysis confirms that TCM for asthenopia research has shown a 45-fold growth in annual publications. Since 2019, the number of English-language literature has also significantly increased, which may be attributed to the sharp increase in the use of video terminals caused by the pandemic and the global recognition of the overall advantages of TCM.

China’s dominance is rooted in its centuries of TCM expertise, abundant clinical resources, and institutional support for integrative medicine. The author and institutional collaboration networks reveal two key patterns: (1) Core clusters drive innovation in mechanism research and therapy optimization; (2) Isolated groups such as regional organizations and small foreign teams reflect an imbalance in cooperation, which hinders the exchange of knowledge and the verification of large-scale effects.

Keyword burst analysis identifies “eye disease” and “acupuncture” as persistent high-intensity keywords, confirming their centrality. “Eye disease” reflects asthenopia’s comorbidity with dry eye or myopia, while “acupuncture” highlights external therapy’s core role. The integration of TCM with modern technology, such as biodegradable microneedles and data mining, further underscores the field’s shift toward precision and evidence-based practice.

### 4.2 New trends

#### 4.2.1 Elucidation of therapeutic mechanisms

The keyword clustering analysis (Figure 7A) identifies #1 iridoid-polyphenolic fraction, #7 novel PLGA, and #8 reducing accommodative lag as core mechanistic themes—directly supported by recent empirical studies:

(1) Iridoid-polyphenolic fraction’s anti-inflammatory effect: Yuan et al. (16) found that iridin (an active component of Belamcanda chinensis, a key source of iridoid-polyphenolic fractions) enhances HCE-2 cell viability and inhibits IL-1β-mediated pyroptosis under hyperosmotic stress, explaining why #1 iridoid-polyphenolic fraction is a core cluster in English literature. This confirms that TCM mechanisms are shifting from macro “liver-kidney regulation” to micro molecular pathways.

(2) Acupuncture’s regulation of ocular accommodation: Chen et al. (17) demonstrated that acupuncture increases dopamine levels in Syrian hamsters via the D1R signaling pathway, suppressing NLRP3 inflammasome activation and reducing accommodative lag, aligning with #8 reducing accommodative lag in English literature. This links TCM’s “meridian unblocking” theory to modern neurobiology, strengthening evidence for acupuncture’s efficacy.

(3) Novel drug delivery systems: The #7 novel PLGA cluster reflects innovations in TCM formulation. For example, Song et al. (18) found that PLGA-based microneedles for sustained acupoint drug release. This addresses TCM’s traditional limitation of poor bioavailability, paving the way for precision external therapy.

These findings confirm that the research on mechanisms is shifting from empirical descriptions to molecular-level verification. This is of vital importance for the promotion of TCM globally and the transformation of its clinical applications.

#### 4.2.2 Therapy optimization of acupoint stimulation modalities in external therapies

The keyword clustering (Figure 7B: #2 thunder-fire noxibustion, #3 press-tack needle) and burst analysis (Figure 9B: “acupuncture and moxibustion” intensity = 2.92) highlight external therapy’s evolution toward precision, personalization, and technological integration. This conclusion is also supported by the institutional cooperation network diagram (Figure 5):

(1) Precision acupoint localization: Some core institutions utilize ultrasound and infrared thermal imaging technologies to accurately identify acupoints, with positioning accuracy reaching the sub-millimeter level (19–21). This improves the consistency of treatment and reduces variations among different operators. It solves the problem of “subjective acupoint selection” traditionally and aligns with the minimally invasive and precise stimulation characteristics of #3 press-tack needle cluster.

(2) Technological integration: The keyword “biodegradable microneedle acupuncture” reflects the integration of traditional acupuncture therapy with nanotechnology. For instance, PLGA micro-needles can release herbal extracts such as chrysanthemum flavonoids at the acupuncture points (18). This approach enhances the therapeutic effect through continuous stimulation and improves patient compliance through this painless drug delivery method, thereby addressing the invasiveness issue of traditional acupuncture.

(3) Standardization: The “randomized controlled trial” cluster indicates that progress has been made in establishing operational norms. For instance, the duration of acupuncture has been standardized at 20–30 min, and the frequency is generally controlled at about 3 times per week (22, 23). This is crucial for the promotion of TCM globally, as it reduces the discrepancies when comparing different studies.

However, the English-language research on external therapies is still relatively limited. The existing studies mostly focus on acupuncture, while there is less exploration of other therapies such as massage and cupping. This highlights the necessity of international cooperation to share the diverse external therapy methods of TCM.

#### 4.2.3 Optimization of herbal decoction protocols for systemic regulation

Chinese literature keyword clustering (Figure 7B: #7 liver-strain, #9 insufficiency of liver blood) and burst analysis (Figure 9B: “liver strain” intensity = 1.93) confirm that herbal decoction optimization focuses on TCM syndrome differentiation and modern formulation technology. It rooted in classical zang-fu theory, such as “liver stores blood, nourishes the eyes” in Huangdi Neijing.

(1) Syndrome differentiation-based personalization: Core teams classify asthenopia into four types and formulated the following decoctions specifically for each type (24–27). For those with liver-kidney deficiency, Qiju Dihuang Pill is used to nourish essence and improve retinal antioxidant enzyme activity (28); for those with liver qi stagnation, Xiaoyao San is taken to soothe the liver and regulate the blood flow in the ocular arteries (29); for those with weak spleen function, Buzhong Yiqi Decoction is used to enhance the spleen function and improve the microcirculation in the eyes (25); for those with heart-blood deficiency, Tianwang Buxin Dan is taken to nourish the mind and reduce eye fatigue caused by mental stress (26). This is in line with the #7 liver-strain cluster, because the liver qi stagnation caused by mental stress in “liver strain” is a major factor in the modern pathogenesis, which demonstrates the adaptability of TCM to contemporary lifestyles.

(2) Modern formulation innovation: The “orthogonal design” burst keyword reflects using statistical methods to optimize herbal ratios. For example, Liu’s Kangyan Pilao Decoction optimized for anti-inflammatory effects via orthogonal testing of 5 herbs (30). Advanced technologies such as microencapsulation and ultrasonic extraction have further enhanced the bioavailability. For instance, Lycium barbarum polysaccharide microspheres can enhance the protective effect on the retina (31).

(3) Combination with Western medicine: The “artificial tears” burst keyword indicates combining TCM decoctions with Western interventions. For example, using iridin plus artificial tears to improve tear film stability, or using self-made Yanggan Runmu decoction combined with sodium hyaluronate on tear secretion and tear inflammatory factors in patients (32), achieving both symptom relief (Western medicine) and root-cause regulation (TCM). This “integrative model” is supported by the #2 low-concentration atropine cluster, where atropine (myopia control) is combined with acupuncture (asthenopia relief) (33–36).

### 4.3 Research hotspots

Through keyword analysis and cluster analysis, we identified two main research focuses, which are closely related to the development of this field toward evidence-based practices and overall comorbidity management.

#### 4.3.1 Evidence-based TCM clinical protocols

The “randomized controlled trial” cluster and “systematic review” burst keyword confirm that evidence-based research is a top priority. Core institutions, such as the Ophthalmology Hospital of the Chinese Academy of Traditional Chinese Medicine, are advancing this via three strategies:

(1) Standardized syndrome differentiation: Expert consensus (7) classifies asthenopia into four TCM patterns (liver-kidney deficiency, spleen qi deficiency, heart-blood deficiency, liver qi stagnation), with validated diagnostic criteria, including tongue coating, pulse condition combined with tear film breakup time and so on (37). This addresses traditional TCM’s subjectivity and enables cross-study comparisons.

(2) Modern efficacy metrics: Clinical studies now integrate objective indicators (e.g., serum IL-6/TNF-α levels for inflammation, macular pigment density for retinal health) with TCM symptom scores. For example, Sun et al. (38) used acupuncture plus lutein to increase macular pigment density, quantifying TCM’s “nourish eye” effect via modern imaging.

(3) Data-driven optimization: The “data mining” burst keyword reflects using big data to identify optimal interventions. Wen et al. (39) used association rule mining to confirm that “Jingming BL1 + Taiyang EX-HN5” is the most effective acupoint combination for video terminal-related asthenopia.

#### 4.3.2 Asthenopia comorbidity models

The “dry eye disease” cluster and “eye disease” burst keyword highlight asthenopia’s comorbidity with ocular and systemic diseases, which aligns with the “holistic regulation” theory of TCM. The analysis identifies three comorbidity patterns:

(1) Metabolic inflammatory axis comorbidities: Asthenopia patients have higher rates of diabetic retinopathy, linked to elevated oxidative stress (IL-6, TNF-α) (40, 41). TCM’s “Phlegm-Stasis Intermingling” theory explains this, using Danshen (Salvia) and Sanqi (Notoginseng) to regulate lipid metabolism and reduce inflammation (42, 43). This aligns with the #0 dry eye syndrome cluster, as dry eye and diabetes share inflammatory pathways (44).

(2) Neuro-ophthalmic psychosomatic networks: Functional MRI reveals reduced prefrontal-visual cortex connectivity in asthenopia patients, correlating with a 30% higher depression rate (45). TCM uses Anshen Yangxin Formula (Ziziphus-Biota Decoction) to modulate the HPA axis and reduce cortisol levels (37), addressing both ocular fatigue and mental stress. This is consistent with the #7 liver-strain cluster, as “liver qi stagnation” links emotional stress to eye symptoms.

(3) Circadian rhythm-mediated disorders: Dysregulated circadian genes (e.g., CLOCK) reduce tear secretion (46), which aligns with TCM’s “Disordered Defense Qi Circulation” theory, meaning that the vital energy cannot function properly, thereby causing dryness in the eyes (47). Herbal fumigation, like chrysanthemum + peppermint, regulates lacrimal secretion via TRPV1 channels (48), which integrates TCM’s “aroma therapy” with modern circadian biology.

### 4.4 Limitations and suggestions

The indicators covered by bibliometrics are contingent upon the temporal span of the analyzed publications. Given the recency of the relevant literature, emergent studies may provide lower values for different indicators, which may lead to distorted results. Furthermore, this study acknowledges several inherent limitations of bibliometric methods, with specific additions as follows:

First, potential bias in the keyword merging process: Although we standardized keywords (e.g., unifying “asthenopia,” “eye fatigue,” “visual fatigue” into “asthenopia”), subjective judgment was involved in determining synonymous terms. For example, “liver-strain” and “liver qi stagnation” were not merged due to subtle differences in TCM connotations (the former emphasizes mental stress, the latter emphasizes qi stagnation), which may have affected the accuracy of clustering results. Future studies could use natural language processing technology to improve keyword standardization objectivity.

Second, challenges of comprehensive search across four databases: Differences in database functionalities (e.g., WOS and PubMed use English search terms, CNKI and Wanfang use Chinese terms) may have led to missed literatures. For example, some regional TCM journals indexed only in CNKI may not have been fully retrieved, and English literature on TCM may have used non-standard terminology (e.g., “Chinese herbs” instead of “Chinese herbal drugs”) that was not captured by our search strings. To mitigate this, we conducted pilot searches to optimize keywords and cross-validated retrieval results across databases, but residual bias cannot be completely excluded.

Third, the “citation lag” phenomenon, where recent publications have not yet accumulated significant citations, may affect the analysis of very recent research trends, potentially underestimating their impact. Finally, the interpretation of keyword clustering, while guided by algorithmic metrics, involves a degree of subjectivity in labeling and defining research themes.

Notably, both the epidemiological patterns of asthenopia and TCM-based preventive/therapeutic strategies constitute dynamic phenomena within an evolving clinical status. This necessitates proactive longitudinal monitoring to establish robust temporal correlations and generate evidence-based insights.

## 5 Conclusion

The integration of TCM in asthenopia management has substantial scholarly value and translational potential. This is due to its millennium-old empirical foundation and alignment with modern precision medicine. The bibliometric analysis identifies three core research directions. (1) Mechanistic exploration: From macro TCM theory to micro molecular pathways; (2) External therapy optimization: Precision acupoint localization, technological integration, and standardization of protocols; (3) Herbal decoction innovation: Syndrome differentiation-based personalization, modern formulation technology, and combination with Western medicine. However, the scattered cooperation among some regions and international teams, as well as methodological deviations, have hindered progress.

Future efforts should focus on: (1) Establishing multinational consortia with harmonized TCM terminology and trial protocols; (2) Using big data and AI to optimize syndrome differentiation and therapy selection; (3) Integrating systemic outcome metrics to fully evaluate TCM’s holistic advantages. With these steps, TCM has the potential to redefine global asthenopia care, offering a complementary approach that addresses both ocular symptoms and underlying systemic imbalances.

## Data Availability

The original contributions presented in this study are included in this article/supplementary material, further inquiries can be directed to the corresponding authors.
